# Nuclear beta-catenin overexpression in metastatic sentinel lymph node is associated with synchronous liver metastasis in colorectal cancer

**DOI:** 10.1186/1746-1596-6-109

**Published:** 2011-11-04

**Authors:** Hongxia Cheng, Hui Liang, Yejun Qin, Ying Liu

**Affiliations:** 1 Department of Pathology, Provincial Hospital Affiliated to Shandong University, Jinan 250021, Shandong, People's Republic of China; 2 Department of otolaryngology, Qianfoshan Hospital Affiliated to Shandong University, Jinan 250014, Shandong, People's Republic of China

**Keywords:** Beta-catenin, Colorectal cancer, Liver metastasis, Sentinel lymph node

## Abstract

**Background:**

Beta-catenin, a component of the Wingless/Wnt signaling pathway, can activate target genes linking with the adenomatous polyposis coli (APC) gene in colorectal cancer. The purpose of this study is to investigate whether nuclear beta-catenin overexpression in metastatic sentinel lymph node(s) [SLN(s)] is associated with synchronous liver metastasis.

**Methods:**

Clinicopathological data from 355 patients (93 cases with liver metastasis and 262 cases without liver metastasis) were reviewed. Beta-catenin expression in metastatic SLN(s) and liver metastatic lesions was examined by immunohistochemistry. The association of nuclear beta-catenin expression in metastatic SLN(s) and liver metastatic lesions was evaluated, and the relationship between nuclear beta-catenin expression and clinicopathological characteristics was analyzed. Finally, univariate and logistic multivariate regression analyses were adopted to discriminate the risk factors of liver metastasis.

**Results:**

Nuclear beta-catenin overexpression in metastatic SLN(s) was observed in 70 patients with liver metastasis and 31 patients without liver metastasis (75.3% vs. 11.8%; P < 0.001). Nuclear beta-catenin expression was noted in all the metastatic lesions. Spearman rank correlation analysis demonstrated that nuclear beta-catenin expression in metastatic SLN(s) had a positive correlation with that in metastatic lesions (r = 0.425, P < 0.001). Univariate and multivariate analyses indicated that nuclear beta-catenin overexpression in metastatic SLN(s) correlated with liver metastasis.

**Conclusions:**

Nuclear beta-catenin overexpression in metastatic SLN(s) is strongly associated with liver metastasis and may contribute to predict liver metastasis.

## Background

Colorectal cancer (CRC) is one of the most common cancers and the second leading cause of cancer-related deaths in the western world, which usually result from uncontrolled metastatic disease. The most common organ metastasis in CRC patients is the liver. For patients with local CRC, the five-year survival rate approaches 90%; however, in metastatic patients, this rate decreases to 19% [1]. The rate of liver metastasis in clinical CRC patients is 5.5%-33.3% [2]. Thus, early detection of liver metastasis in CRC is especially important to improve patient survival rate. Several studies have investigated the risk factors influencing liver metastasis [3-7]. Histopathologically, the presence of venous invasion [8,9], a deeper level of invasion, less differentiated carcinoma cells and the presence of lymph node metastasis have been reported to be risk factors [10]. The biological factors related to liver metastasis have been identified as EGF [6], Arp2 [5], TGF-α [11], CD44 [12], and CD10 [13,14].

It has been suggested that CRC might show cellular dedifferentiation in the invasive area, with loss of an epithelial phenotype and a gain of a mesenchymal phenotype, which facilitates invasive and metastatic growth of originally differentiated cancer cells. The malignant progression is called an epithelial-mesenchymal transition (EMT) [15-17]. One of the key oncogenic proteins that might drive EMT in colorectal carcinogenesis is beta-catenin [18]. Its localization relates to its function in cancer growth [19]. Beta-catenin in cytoplasm and membrane binds with the intracellular domain of E-cadherin, which is a cell-to-cell adhesion molecule, and then plays a significant role in maintaining normal tissue architecture. Nuclear beta-catenin associates with members of the TCF/LEF family of transcription factors [20] and acts as a transcriptional activator of numerous target genes, exerting predominantly tumor-promoting functions. Increased nuclear beta-catenin has been shown to correlate with liver metastasis in CRC [21].

The impact of sentinel lymph node biopsy (SLNB) has been profound in the treatment of melanoma [22,23] and breast carcinoma [24,25]. Over the past decade, numerous studies have investigated this technology in other diseases including gastrointestinal carcinoma [26]. SLNB in CRC is investigational and the potential impact of this technique on the overall treatment of this disease could be significant [27]. In CRC, the lymphatic drainage proceeds from the submucosal lymphoid follicles through the bowel wall to the epicolic, paracolic, and finally, the para-aortic nodes [28]. The SLN(s) are the first to receive lymphatic drainage from the primary tumor. They also have the greatest chance of harboring metastasis when the disease has metastasized to the regional lymphatic basin. Focused pathologic examination of SLN(s) has been used to efficiently search for metastatic disease that may not be identified by standard histopathologic methods. Previous studies demonstrated that SLN mapping and immunohistochemical diagnosis of SLN micrometastases can improve staging accuracy in CRC, and intraoperative diagnosis of SLN micrometastases is crucial for planning the operation and the determination of adjuvant therapy [29]. A recent report indicated that increased expression of hnRNP A1, Ezrin, tubulin beta-2C, and Annexin A1 in metastatic SLN(s) suggests a significantly elevated early CRC metastasis [30].

Although the association of nuclear beta-catenin expression in CRC with liver metastasis has been reported, the previous study focused mainly on beta-catenin expression in primary tumors. Correlation between nuclear beta-catenin expression in metastatic SLN(s) and matched liver metastatic lesions is unknown. Herein, the nuclear beta-catenin expression in metastatic SLN(s) and matched liver metastatic lesions was investigated to identify the relationship between both expressions. Meanwhile, the relationship between nuclear beta-catenin expression and clinicopathological characteristics was analyzed. Finally, univariate and logistic multivariate regression analyses were adopted to discriminate the risk factors of liver metastasis. In this study, nuclear beta-catenin overexpression in SLN(s) was shown to be associated with synchronous liver metastasis. This study is the first to elucidate this relationship based on clinical and pathological data. The clinical significance of this research is that the provision of a promising predictor of liver metastasis in CRC.

## Methods

### Study population and data collection

This study cohort comprised 355 CRC patients with metastatic SLN(s) who underwent treatment in the Provincial Hospital and the Qianfoshan Hospital affiliated to Shandong University from March 2001 to March 2009. There were 247 men and 108 women. Their mean age was 65 years, ranging from 35 to 85 years. Of the 355 cases, 93 had synchronous liver metastasis. Tumor stage was based on the American Joint Committee on Cancer TNM staging system (6th edition, 2002) [31]. The inclusion criteria were as follows: (1) patients underwent surgical treatment and were histologically identified as having CRC and liver metastasis; (2) liver metastasis was identified within six months after finding the primary tumor; and (3) patients with no prior history of liver-directed treatment and radiofrequency ablation. The exclusion criteria were as follows: (1) liver metastasis arose after surgical treatment; (2) patients accepted other treatment simultaneously, such as chemotherapy; (3) hereditary non-polyposis colorectal cancer patients and patients with familial adenomatous polyposis; (4) patients with other liver diseases, such as liver cirrhosis; and (5) patients with extrahepatic metastasis. All 355 patients fulfilled the inclusion criteria. On the other hand, 23 cases whose liver metastasis arose after surgical treatment, 11 cases who accepted chemotherapy, 1 case of familial adenomatous polyposis, 2 cases with liver cirrhosis and 5 cases with extrahepatic metastases were excluded. This study was approved by Ethics Committee of Provincial Hospital and Qianfoshan Hospital. Clinical information related to diagnostic procedures and clinicopathological characteristics were collected from medical records.

### Ex vivo sentinel node mapping technique

Ex vivo sentinel node mapping was performed as described previously [32]. Briefly, within 10 min of resection, colorectal specimens were divided along their anti-mesenteric border with a scalpel, thereby exposing the tumor with approximately 4 cm of normal mucosa proximally and distally. Next, 1-1.5 mL Lymphazurin (Isosulfan blue dye; Tyco, OH) was injected with a tuberculin syringe quadrantically around the tumor. This was performed subserosally if the tumor was above the peritoneal reflection, or submucosally if below it, because the bowel lacks serosa below the peritoneal reflection. Injection areas were gently massaged for 2-3 min to generate the flow of dye along the lymphatics. The first 1-4 lymph nodes stained blue were designated SLN(s). The SLN specimen was then placed directly into formalin. Two designated pathologists performed routine pathologic evaluation 24-72 hours later.

### Immunohistochemistry

The specimens from patients were embedded in paraffin and cut into sections for immunohistochemical staining. Informed consent for the use of the specimens was obtained from all patients. Immunohistochemical staining was performed with the streptavidin peroxidase complex method. Tissue sections of 4 μM thickness were deparaffinized and microwaved for 15 min, twice in 10 mM citrate buffer (pH 6.0) at 100°C to retrieve the antigens, followed by incubation in 3% H2O2 for 10 min to quench the endogenous peroxidase. Nonspecific binding of antibodies was inhibited by incubation in 5% normal goat serum for 20 min in a humid chamber. Tissue sections were then incubated with mouse monoclonal anti-beta-catenin antibody (diluted 1:50, mouse IgG1; Cell Signaling Technology, Boston, USA) overnight at 4°C. After three washes with PBS, tissue sections were incubated with biotinylated goat anti-mouse IgG for 30 min at room temperature. After washing, slides were incubated in streptavidin-peroxidase complex for 20 min at 37°C, washed three times, visualized using DAB, and counterstained with hematoxylin. As a negative control, sections were stained without the addition of a primary antibody.

As for the immunohistochemistry assessment, six sights were selected randomly and observed at × 400 magnification by two pathologists. The immunostained slides were scored as described previously [33]. Briefly, the immunostained slides were scored using the sum of signal intensity (0 = no expression; 1 = weak expression; 2 = moderate expression; 3 = strong expression) and the percentage of positive cells (% tumor cells: 0 = 0%; 1 = 1%-25%; 2 = 26%-50%; 3 = 51%-75%; and 4 = 76%-100%). If a moderate or strong expression was observed in the nuclei, then nuclear beta-catenin immunoreactivity in SLN(s) was regarded as positive. Nuclear beta-catenin overexpression was defined as a positive nuclear beta-catenin expression in > 50% of tumor cells. This cutoff value was chosen after a preliminary quantification because it was close to the mean value.

### Statistical analysis

Statistical analysis was carried out using SPSS 16.0. Spearman rank correlation analysis was used to evaluate the relationship of nuclear beta-catenin expression in metastatic SLN(s) and liver metastatic lesions. The χ2 and Fisher exact tests were adopted to analyze the correlation between nuclear beta-catenin expression and clinicopathological characteristics. Finally, univariate and logistic multivariate regression analyses were adopted to discriminate the independent risk factors of liver metastasis. P < 0.05 was considered statistically significant.

## Results

### Nuclear beta-catenin expression in SLN(s) and liver metastatic lesions

**Figure 1:**
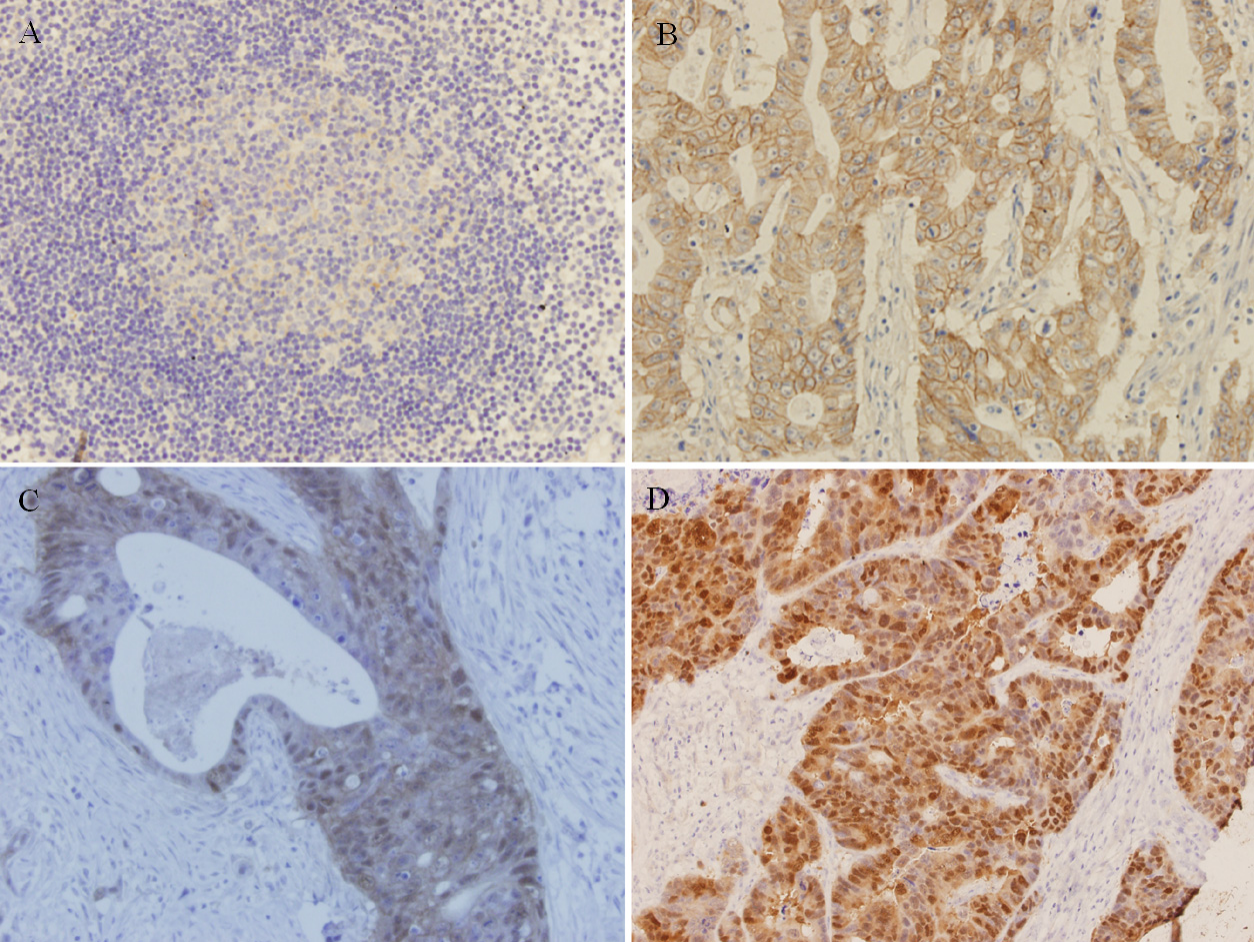
**Beta-catenin expression in metastatic SLN(s) and liver metastatic lesions (×400)**. (A) Normal SLN (B) beta-catenin presents mostly in a membrane-associated staining pattern in metastatic SLN(s) in patients without liver metastasis (C) Nuclear beta-catenin is overexpressed in metastatic SLN(s) in patients with liver metastasis (D) Nuclear beta-catenin expression in liver metastatic lesions.

A total of 777 metastatic lymph nodes from 355 patients were excised and processed. The mean number in each patient was 2.4 ± 1.3. Beta-catenin expression was observed in all the SLN specimens. Nuclear beta-catenin expression was observed in metastatic SLN(s) in 101 patients. In patients without liver metastasis beta-catenin presented mostly in a membrane-associated staining pattern (Figure 1B). Nuclear beta-catenin overexpression in metastatic SLN(s) was observed in 31 cases (11.8%). In patients with liver metastasis, nuclear beta-catenin overexpression in metastatic SLN(s) could be observed in 70 cases (75.3%) (Figure 1C). Nuclear beta-catenin overexpression in metastatic SLN(s) was more evident in patients with liver metastasis than in patients without liver metastasis (P < 0.001). On the other hand, nuclear beta-catenin expression was noted in all the metastatic lesions (Figure 1D). Spearman rank correlation analysis indicated that nuclear beta-catenin expression in metastatic SLN(s) had a positive correlation with that in metastatic lesions (r = 0.425, P < 0.001) (Table 1).

**Table 1:** Correlation between nuclear beta-catenin expression in SLN(s) and the matched liver metastatic lesions

beta-catenin expression in SLN(s)	beta-catenin expression in liver metastatic lesions				Total
		
	1-25%	26-50%	51-75%	76-100%	
1-25%	0	2	0	0	2
26-50%	0	9	11	1	21
51-75%	0	0	37	8	45
76-100%	0	0	20	5	25
Total	0	11	68	14	93

### Relationship between nuclear beta-catenin expression in SLN(s) and clinicopathological characteristics

The χ2 test was performed to investigate the relationship between nuclear beta-catenin expression in metastatic SLN(s) and clinicopathological features. Beta-catenin expression in metastatic SLN(s) was significantly different for age (P < 0.001), type of tumor (P < 0.001), tumor cell differentiation (P < 0.001), tumor invasion depth (P < 0.001), lymph node metastasis (P = 0.003), liver metastasis (P < 0.001) and TNM stage (P < 0.001) (Table 2).

**Table 2:** Correlation of nuclear beta-catenin expression in SLN(s) with clinicopathological characteristics

Characteristics	Nuclear beta-catenin expression			P*
		
	n	≤ 50%	> 50%	
Gender				
Male	247	178	69	0.745
Female	108	76	32	
Age				
≤ 55	54	15	39	< 0.001
> 55	301	239	62	
Tumour localization				
Rectum	203	145	58	0.954
Colon	152	109	43	
Tumour size (cm)				
≤ 5	120	90	30	0.303
≤ 5	235	164	71	
Type of tumour				
Adenocarcinoma	299	221	78	< 0.001
Mucinous adenocarcinoma	31	11	20	
Signet-ring cell carcinoma	16	14	2	
Undifferentiated carcinoma	9	8	1	
Tumour cell differentiation				
Well/moderately differentiated	279	218	61	< 0.001
Poorly differentiated	76	36	40	
Tumour invasion depth				
T_1_	5	5	0	< 0.001
T_2_	63	56	7	
T_3_	252	176	76	
T_4_	35	17	18	
Lymph node metastasis				
< 4	123	100	23	0.003
≥ 4	232	154	78	
Liver metastasis				
Negative	262	231	31	< 0.001
Positive	93	23	70	
TNM stage				
I	5	5	0	< 0.001
II	20	20	0	
III	237	206	31	
IV	93	23	70	

* *P *value is the result of χ^2 ^test in the same factor, and < 0.05 is considered statistically significant.

### Nnuclear beta-catenin overexpression in SLN(s) is an independent risk factor of synchronous liver metastasis

To discriminate independent the risk factors of synchronous liver metastasis, clinicopathological characteristics were divided into two groups according to liver metastasis. Univariate analysis and logistic multivariate regression model were adopted to discriminate the risk factors of liver metastasis. The χ2 test demonstrated that liver metastasis is associated with age (P < 0.001), tumor size (P = 0.001), type of tumor (P = 0.002), tumor cell differentiation (P < 0.001), tumor invasion depth (P = 0.002), lymph node metastasis (P = 0.001) and nuclear beta-catenin overexpression in metastatic SLN(s) (P < 0.001) (Table 3). However, logistic multivariate regression analysis retained only six factors in the model (Table 4). These factors are age (P = 0.004), tumor size (P = 0.002), tumor invasion depth (P < 0.001), tumor cell differentiation (P = 0.023), lymph node metastasis (P = 0.002) and nuclear beta-catenin overexpression in metastatic SLN(s) (P < 0.001).

**Table 3:** Clinicopathological risk factors classified according to liver metastasis

Risk factors	Liver Metastasis			P*
		
	n	Negative	Positive	
Gender				
Male	247	182	65	0.939
Female	108	80	28	
Age				
≤ 55	54	22	32	< 0.001
> 55	301	240	61	
Tumour localization				
Rectum	203	150	53	0.965
Colon	152	112	40	
Tumour size (cm)				
≤ 5	120	102	18	0.001
> 5	235	160	75	
Type of tumour				
Adenocarcinoma	299	232	67	0.002
Mucinous adenocarcinoma	31	17	14	
Signet-ring cell carcinoma	16	9	7	
Undifferentiated carcinoma	9	4	5	
Tumour cell differentiation				
Well/moderately differentiated	279	236	43	< 0.001
Poorly differentiated	76	26	50	
Tumour invasion depth				
T_1_	5	5	0	0.002
T_2_	63	20	1	
T_3_	252	221	72	
T_4_	35	25	20	
Lymph node metastasis				
< 4	123	104	19	0.001
≥ 4	232	158	74	
Nuclear beta-catenin expression in SLN(s)				
≤ 50%	254	231	23	< 0.001
> 50%	101	31	70	

* *P *value is the result of χ^2 ^test in the same factor, and < 0.05 is considered statistically significant.

**Table 4:** Variables in the equation for the multivariate logistic regression model

Risk factors	*B*	S.E	Wald	D*f*	**Sig**.	Exp(*B*)
Nuclear beta-catenin in SLN(s)	1.787	0.316	23.744	1	< 0.001	9.118
Tumour invasion depth	1.635	0.365	15.533	1	< 0.001	7.213
Tumour size	1.372	0.413	8.435	1	0.002	5.317
Lymph node metastasis	1.368	0.482	8.173	1	0.002	4.759
Age	1.317	0.471	7.456	1	0.004	3.337
Tumour cell differentiation	1.214	0.544	4.739	1	0.023	2.458

## Discussion

To investigate whether nuclear beta-catenin overexpression in metastatic SLN(s) is correlated with synchronous liver metastasis, beta-catenin expression in metastatic SLN(s) and matched liver metastatic lesions in 355 CRC patients were examined, and their clinicopathological data were analyzed retrospectively. Nuclear beta-catenin overexpression in SLN(s) has been demonstrated to be associated with synchronous liver metastasis. According to our knowledge, this study is the first to elucidate the relationship between nuclear beta-catenin overexpression in SLN(s) and synchronous liver metastasis using clinical and pathological data. The clinical significance of the current study is the provision of a promising predictor of liver metastasis in CRC.

Beta-catenin is a key component of adherens junctions, which are necessary for homophilic cell-cell adhesions [34]. Membranous beta-catenin determines the epithelial phenotype; nuclear beta-catenin represents a transcriptional regulator and is a main effector of the Wnt signaling pathway [35]. A recent study indicated that the expression of E-cadherin-catenin complex in SLN in breast carcinoma is related to tumor morphology [36]. In the current study, only those with synchronous liver metastasis were included to exclude the influence of treatments such as operative procedures and chemotherapy. Our data demonstrated that nuclear beta-catenin overexpression in metastatic SLN(s) is more evident in patients with liver metastasis than in patients without liver metastasis. This finding suggests that nuclear beta-catenin overexpression in metastatic SLN(s) correlates with synchronous liver metastasis. In addition, a nuclear beta-catenin expression was observed in the matched liver metastatic lesions, which had a positive correlation with nuclear beta-catenin expression in the metastatic SLN(s). All the above-mentioned immunohistochemical data indicated that the accumulation of nuclear beta-catenin in metastatic SLN(s) may correlate with the formation of liver metastatic lesion in CRC.

With regard to correlation between nuclear beta-catenin in metastatic SLN(s) and clinicopathological characteristics, no previous report was found. However, previous investigators reported contradictory results for the relationship between nuclear beta-catenin in CRC and clinicopathological characteristics. Zhang et al. believed that nuclear beta-catenin accumulation is related to tumor stage and/or metastasis [37]. However, correlations between nuclear beta-catenin and important clinicopathological variables were not observed in Baldus's study [38]. Our data demonstrated that nuclear beta-catenin expression in metastatic SLN(s) is associated with age, type of tumor, tumor cell differentiation, tumor invasion depth, lymph node metastasis, liver metastasis and TNM stage. This result indicates that nuclear beta-catenin overexpression is related to an advanced tumor stage and poor tumor cell differentiation, which may be helpful for explaining the association between nuclear beta-catenin overexpression in metastatic SLN(s) and liver metastasis. As for age, controversy remains about the differences in CRC between younger and older patients. Some researchers indicated no differences in relation to the major histopathological features and the pathologic grade or stage in younger CRC patients compared with older patients [39,40]. On the other hand, some studies reported young CRC patients were more likely to be characterized with poor differentiation and higher grade, later stage (III/IV), and poor prognosis [41,42]. Our results are mainly consistent with the latter finding. In our research, patients younger than 55 years had a higher incidence rate of liver metastases, and nuclear beta-catenin overexpression in metastatic SLN(s) was more evident. Thus, these data may also suggest the association between advanced tumor and nuclear beta-catenin overexpression, and the close association of beta-catenin overexpression in metastatic SLN(s) and age with liver metastasis. The low suspicion for CRC and the low rate of screening colonoscopy in younger patients, which may directly cause delay in making the right diagnosis, may be the main reasons for the different research results. In the present study, we did not find significance between gender and nuclear beta-catenin overexpression in metastatic SLN(s) or liver metastasis.

Univariate analysis demonstrated that nuclear beta-catenin overexpression in metastatic SLN(s) is associated with liver metastasis, which coincides with the result of immunohistochemical analysis. To obtain a more precise estimate of the influence of nuclear beta-catenin overexpression in metastatic SLN(s) on liver metastasis, a multivariate logistic regression analysis model was constructed to discriminate the independent risk factors of synchronous liver metastasis. Nuclear beta-catenin overexpression in metastatic SLN(s) remains in the model (Table 4), suggesting that nuclear beta-catenin overexpression in metastatic SLN(s) may influence liver metastasis independently. The current TNM staging system for CRC has been useful in predicting the outcome after definitive resection, but its potential value to predict distant metastasis is limited. Attempts have been made to predict tumor behavior better by identifying novel biologic prognostic factors. Although previous studies have indicated that nuclear beta-catenin expression was associated with liver metastasis, the proof was limited to the immunohistochemical analysis of primary tumor. In the current study, nuclear beta-catenin overexpression was found not only in the metastatic SLN(s), but also in the matched metastatic liver lesions. Moreover, the association between nuclear beta-catenin overexpression and liver metastasis was verified by clinical data. Nuclear beta-catenin expression in metastatic SLN(s) may become a possible clinically useful marker to distinguish between highly metastatic CRC and less aggressive CRC.

In this study, only the association of nuclear beta-catenin overexpression in metastatic SLN(s) with synchronous liver metastases was analyzed. To exclude the influence of treatments on liver metastasis, liver metastases that occurred after surgical treatments were not examined. Investigating whether the factors associated with synchronous metastasis also play an important role in metastasis that occurs after surgery is also a very useful direction to pursue. Intriguingly, 31 cases showed nuclear beta-catenin overexpression in metastatic SLN(s) in patients without synchronous liver metastases. Examining if liver metastases would occur more frequently in these cases than in those without overexpression would be significant. These cases are under follow-up, and the same type patients are being gathered. Further studies are necessary to evaluate the relationship between nuclear beta-catenin overexpression in metastatic SLN(s) and liver metastases occurring after surgery in CRC.

## Conclusions

Our data demonstrated that nuclear beta-catenin overexpression in metastatic SLN(s) is associated with synchronous liver metastasis, while nuclear beta-catenin expression in liver metastatic lesions is related to that in metastatic SLN(s). Both clinical and pathological data authenticate our conclusion. Nuclear beta-catenin overexpression in metastatic SLN(s) in CRC may be used as a valuable predictor of liver metastasis. Clinical surveys with larger study cohorts will be needed to verify our findings.

## Competing interests

The authors declare that they have no competing interests.

## Authors' contributions

HC designed the study and analyzed the data. HC and YQ reviewed all the pathological slides. HL performed Ex vivo sentinel node mapping. HC and YL did the immunohistochemical analysis. All authors read and approved the final manuscript.
